# Ladies and the Journal

**Published:** 1875-04

**Authors:** 


Can't some of the good ladies who grace the hearthstones and adorn the homes of our brethren take a useful hint from the following:

"My wife says I ought by all means to pay for the Journal, even for the comfort she has derived from it. For the last ten or twelve years she has been most painfully afflicted with an ingrowing toe-nail. In the October number of your Journal I found the suggestion of its treatment by applying the muriated tincture iron to the nail and surrounding diseased structures. Several weeks ago she began the new treatment, and has already the satisfaction to realize that she is almost completely cured, for which she feels greatly indebted," etc.

We have reason to know that very many of the good ladies watch with interest the monthly visits of our Journal, and we beg to assure them that they are not forgotten in the use of our editorial scissors. May we not invoke their more retentive memories to jog the elbow of the good man of the house in due season when the subscription runs out.

